# Chronic oral administration of minocycline to sheep with ovine CLN6 neuronal ceroid lipofuscinosis maintains pharmacological concentrations in the brain but does not suppress neuroinflammation or disease progression

**DOI:** 10.1186/1742-2094-10-97

**Published:** 2013-07-30

**Authors:** Graham W Kay, David N Palmer

**Affiliations:** 1 Faculty of Agriculture and Life Sciences, Lincoln University, PO Box 85084, Lincoln 7647, New Zealand

**Keywords:** Anti-inflammatory drugs, Batten disease, Cerebrospinal fluid, Cortical atrophy, CSF, HPLC, NCL, Neurodegeneration, Neuroinflammation, Ovine model

## Abstract

**Background:**

The neuronal ceroid lipofuscinoses (NCLs; or Batten disease) are fatal inherited human neurodegenerative diseases affecting an estimated 1:12,500 live births worldwide. They are caused by mutations in at least 11 different genes. Currently, there are no effective treatments. Progress into understanding pathogenesis and possible therapies depends on studying animal models. The most studied animals are the CLN6 South Hampshire sheep, in which the course of neuropathology closely follows that in affected children. Neurodegeneration, a hallmark of the disease, has been linked to neuroinflammation and is consequent to it. Activation of astrocytes and microglia begins prenatally, starting from specific foci associated with the later development of progressive cortical atrophy and the development of clinical symptoms, including the occipital cortex and blindness. Both neurodegeneration and neuroinflammation generalize and become more severe with increasing age and increasing clinical severity. The purpose of this study was to determine if chronic administration of an anti-inflammatory drug, minocycline, from an early age would halt or reverse the development of disease.

**Method:**

Minocycline, a tetracycline family antibiotic with activity against neuroinflammation, was tested by chronic oral administration of 25 mg minocycline/kg/day to presymptomatic lambs affected with CLN6 NCL at 3 months of age to 14 months of age, when clinical symptoms are obvious, to determine if this would suppress neuroinflammation or disease progression.

**Results:**

Minocycline was absorbed without significant rumen biotransformation to maintain pharmacological concentrations of 1 μM in plasma and 400 nM in cerebrospinal fluid, but these did not result in inhibition of microglial activation or astrocytosis and did not change the neuronal loss or clinical course of the disease.

**Conclusion:**

Oral administration is an effective route for drug delivery to the central nervous system in large animals, and model studies in these animals should precede highly speculative procedures in humans. Minocycline does not inhibit a critical step in the neuroinflammatory cascade in this form of Batten disease. Identification of the critical steps in the neuroinflammatory cascade in neurodegenerative diseases, and targeting of specific drugs to them, will greatly increase the likelihood of success.

## Background

The neuronal ceroid lipofuscinoses (NCLs; or Batten disease) are a group of fatal inherited human neurodegenerative diseases affecting about 1:12,500 live births worldwide [1]. NCLs are caused by over 360 mutations in at least 8 different genes responsible for childhood forms [2] and another 3 recently suggested to be associated with adult forms [3-5] (see http://www.ucl.ac.uk/ncl/). Affected children become blind, subject to fits and seizures and bed-ridden, and they die prematurely. Currently, there are no effective treatments for any form of the disease. Various naturally occurring or engineered animal models of the human diseases exist, the most studied large animal model being the well-characterized CLN6 South Hampshire sheep model [6]. As in most forms of NCL, subunit c of mitochondrial ATP synthase accumulates in lysosome-derived organelles in most cells, but the biochemical mechanism of this storage is unknown [7-10].

The strength of this model is that the disease progression and course of neuropathology in affected lambs is similar to that in affected children. Affected lambs are apparently normal at birth but become blind from 10 to 14 months of age and decline until mature disease when aged about 2 years. Neurodegeneration, a hallmark of the disease, has been linked to neuroinflammation and is consequent to it. Activation of astrocytes and microglia precedes the loss of cortical neurons and progressive cortical atrophy, starting from particular neocortical foci and becoming widespread in more advanced disease [11,12]. This results in the collapse of layers, particularly layers II to IV. Glial activation is observed perinatally in affected sheep, with reactive perivascular astrocytes being observable in the first trimester, reactive astrocytes observable in the gray matter in the third trimester and activated microglia become apparent shortly after birth [13]. Brain atrophy is not apparent until 4 to 6 months after birth, and clinical symptoms do not develop until some months later, suggesting a potential window for anti-inflammatory therapy after birth and before neurodegeneration sets in.

Chronic inflammation is thought to be detrimental to neurons [14-16], and neuroinflammation has also been indicated as a contributing factor to various other neurodegenerative conditions [17], including multiple sclerosis, Alzheimer disease [18], Parkinson disease [19], HIV-associated dementia, scrapie, trauma and ischemia. A possible therapeutic option is treatment with minocycline, a second-generation antibiotic belonging to the tetracycline family, which also has anti-inflammatory activity, including activity against neuroinflammation [20,21], and targets microglia [22].

Minocycline is easily absorbed when administered orally and crosses the blood–brain barrier readily. Treatment trials in both animal models and human patients have been carried out for a number of neurodegenerative diseases, including multiple sclerosis, spinal cord injury, amyotrophic lateral sclerosis, Huntington disease, Parkinson disease and Alzheimer disease, with encouraging results in a number of cases. For example, recent rodent model trials have indicated efficacy against hypoxic-ischemic brain injury [23], blast-induced traumatic brain injury [24], Alzheimer disease-like amyloid pathology [25] and Huntington disease-like symptoms [26]. However, results from rodent model studies have not been very good indicators of human studies, indicating that larger animal models would be preferred, and there have also been some concerns about chronic treatment in some cases [20,21]. Given the interaction of glial activation and neurodegeneration in the NCLs outlined above, minocycline is an obvious therapeutic candidate to be explored for treatment of the NCLs. The sheep affected by CLN6 provide a good model for this investigation, but as they are ruminants with a very active microbial rumen flora, two additional factors need to be considered, namely, the effect of the antibacterial activity on rumen function and possible rumen biotransformation of minocycline that might affect the penetration of the drug into the sheep plasma and cerebrospinal fluid (CSF).

The aims of this investigation were to determine conditions that allowed chronic administration of minocycline to a ruminant, find out if pharmacologically effective plasma and CSF concentrations of minocycline could be obtained and ascertain whether these would affect glial inflammation and the course of neurodegeneration in CLN6 Batten disease.

## Materials and methods

### Sheep

South Hampshire heterozygous carrier ewes were mated to homozygous affected rams and lambs genotyped as carrier or affected using the disease-associated A/G polymorphism [6]. All animals used were healthy and apparently clinically normal at the commencement of the trials. They were housed indoors in individual pens and fed a balanced commercial pelleted ration (800 g/day) and lucerne chaff (400 g/day). Age-matched heterozygous sheep were used as controls. All procedures were approved by the Lincoln University Animal Ethics Committee in compliance with the New Zealand Animal Welfare Act (1999) and in accordance with US National Institutes of Health guidelines.

### Minocycline administration and animal monitoring

Minocycline hydrochloride (100 to 150 g; Archimica S.r.L., Origgio, Italy) was dissolved in 100 ml of dimethyl sulfoxide, then diluted in 30% animal feed grade molasses to 200 mg/ml. Aliquots were stored at −20°C. This solution was sprayed onto daily rations to deliver the required dose.

In the therapy trial, three 3-month-old affected rams began receiving minocycline in molasses at 2 mg/kg/day and two controls received molasses only. The minocycline dosage was increased stepwise to 25 mg/kg/day over 6 weeks, which was maintained for a further 5 months, then lowered to 16 mg/kg/day for a further 4 months, after which the animals were killed.

Animals were weighed weekly during their growth phase and then at 2- to 3-week intervals, and minocycline dosages adjusted accordingly. Every 6 to 8 weeks, sheep were anesthetized with intravenously administered 1:1 ketamine:diazepam (ketamine, Phoenix Ketamine, Auckland, NZ; and diazepam, Pamlin, Parnell Laboratories, East Tamaki, NZ) before taking cerebrospinal fluid (CSF) samples from the cisterna magnum, jugular blood samples and computed tomography (CT) scans for brain volume determinations. After 3 months of drug administration, possible liver damage was assessed from the serum activities of γ-glutamyltransferase and glutamate dehydrogenase, determined by Gribbles Veterinary Pathology, Auckland, NZ.

### Minocycline high-performance liquid chromatography

**Figure 1:**
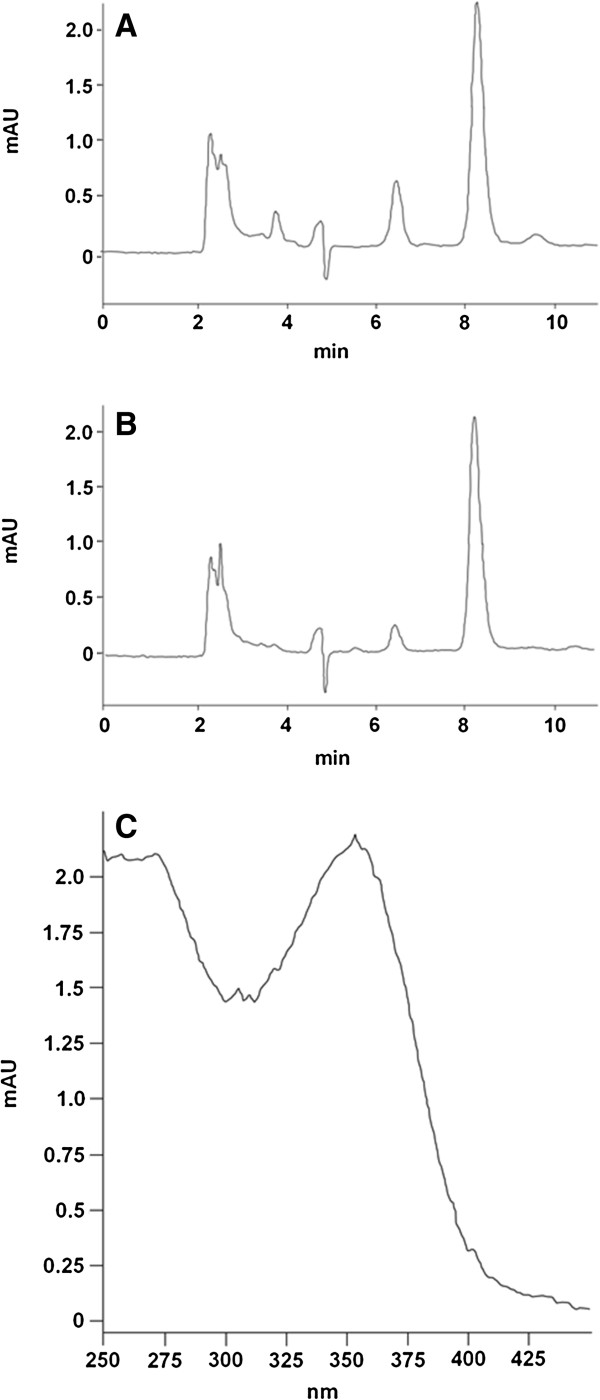
**High-performance liquid chromatography of minocycline.** High-performance liquid chromatography elution of minocycline extracted from plasma **(A)** and cerebrospinal fluid **(B)** taken from the same animal after chronic treatment with minocycline at 25/kg/day. The minocycline peak eluted consistently between 8.2 and 8.3 min. Scanning this peak from 250 to 450 nm yielded a single absorbance peak at 355 nm **(C)**.

Minocycline extracted from plasma and CSF was analyzed by high-performance liquid chromatography (HPLC) [27]. Aliquots (500 μl) of plasma, CSF and standards (0.08 to 10.12 μM minocycline in plasma or CSF) were diluted with 750 μl of sodium phosphate sulfite buffer (2.4 M disodium hydrogen phosphate, 4.0 M sodium sulfite, pH 6.5) and thoroughly mixed with 4 ml of ethyl acetate. After centrifugation to separate the phases, the aqueous phase was frozen and the organic phase was poured off into 50 μl of 0.2% ascorbic acid and 0.1% cysteine in methanol. Samples were dried at 39°C under nitrogen, and the residue was dissolved in 250 μl of running buffer, an 85:12:3 mixture of a solution of 0.1 M potassium nitrite, 0.05 M citric acid and 0.033 M sodium citrate:dimethylformamide:ethyl acetate. After centrifugation, 10,000 rpm, 2 min, 50-μl aliquots were injected onto a Brava BDS C8 column W 100 (4.6 mm × 3 μm; Grace Davison Discovery Sciences, Deerfield, IL, USA) in an Agilent HPLC fitted with a diode display array detector (Agilent 1100 Series; Agilent Technologies, Walbronn, Germany) and eluted with running buffer at 0.5 ml/min. Minocycline, retention time 8.2 to 8.4 min, was detected at 350 nm (Figure 1). The mean ± SD extraction efficiency for minocycline was 64.2 ± 4.5%. All elution peaks with a retention time of 6 min or more were scanned from 250 to 450 nm, and the spectra were compared to plasma and CSF standards to detect any minocycline metabolites.

### Clinical assessment of vision

Vision was scored weekly by awareness of hand movement on a scale of apparent normal awareness = 15, subnormal awareness = 10, marginal awareness = 5 and no awareness = 0.

### Assessment of brain atrophy

CT scans were performed under anesthesia in a GE ProSpeed CT Scanner (GE Healthcare, Hyogo, Japan). Coronal slices 1 mm thick were scanned at 5-mm intervals, 120 kV, 160 mA, 2 s, and brain volumes were determined by using the Cavalieri method from the areas of each slice. Volumes from the experimental animals, determined every 6 to 8 weeks, were compared with those of a cohort of normal, unaffected sheep.

### Assessment of brain pathology

Brains were perfusion fixed in 10% formalin immediately post-mortem, divided down the midline, equilibrated in cryoprotectant solution (10% ethylene glycol and 30% sucrose in saline) at 4°C and stored frozen. Serial sagittal sections 50 μm thick were cut on a sliding freezing microtome (Microm GmbH, Walldorf, Germany) and stored individually at –20°C in 100 μl of cryoprotectant in 96-well plates as described previously [12,13]. Nissl staining and immunostaining were done on floating sections. Microglia and astrocytes were detected using biotinylated α-D-galactose-specific isolectin I-B4 from *Griffonia simplicifolia* (GSB4; Vector Laboratories, Peterborough, UK) and anti-glial fibrillary acidic protein (anti-GFAP; Dako, Carpinteria, CA, USA), respectively, and diluted in 0.2% Triton X-100 in phosphate-buffered saline (PBST) containing 1% normal goat serum. Biotinylated anti-rabbit secondary antibody (Sigma-Aldrich, St Louis, MO, USA) was diluted 1:1,000 in the same buffer. Sections were washed three times with PBST solution containing 1% normal goat serum for 3 h, and antibody and lectin binding were detected using horseradish peroxidase (ExtrAvidin; Sigma-Aldrich) and diaminobenzidine tetrahydrochloride in 0.1 M acetate buffer at pH 6.0.

Layer thickness measurements on the brain sections were determined using SPOT software (SPOT Imaging Solutions, Sterling Heights, MI, USA). All analyses and images were of the visual cortex and occipital lobe, the regions first and most affected by neurodegeneration [12]. Measurements were made from the pial surface of the cortex to the layer V–VI boundary since sheep layer VI is difficult to define in affected brains and measurements from layers I to V cover the entire gray matter apart from this difficult-to-see layer. The distances from layers II to V were also measured since layer I is the least affected by neurodegeneration [12] so comparisons of layers II to V offer the most sensitive measure of any protective effect of minocycline on neuronal layers.

GSB4 and GFAP immunoreactivities in the cortical region encompassing neuronal layers II to V in sections of the visual cortex of affected treated and untreated animals were captured for image analysis by using a Nikon Digital Sight DSFi1 camera attached to a Nikon Eclipse 50*i* microscope (Nikon Instruments, Tokyo, Japan). Data were analyzed after threshold analysis using NIS-Elements Advanced Research version 3.0 software (Nikon Instruments). Three different fields per section per animal were analyzed (pixel classifier), and the results are expressed as percentage per unit area.

## Results

### Drug delivery

An effective dosing regimen was developed that gave the rumen flora time to adjust to the antibiotic activity of minocycline and resulted in pharmacologically sensible plasma and CSF concentrations of untransformed minocycline. This regimen involved increasing dietary minocycline from an initial dose of only 2 mg/kg/day. A higher initial dosing of 4 mg/kg/day caused a sharp drop in feed intake and was halted. For the therapy trial, three 3.4-month-old affected ram lambs accustomed to molasses began receiving minocycline at 2 mg/kg/day. The dosage was then increased stepwise to 25 mg minocycline/kg/day at 4.5 months of age, which was maintained for 5 months and then lowered to 16 mg/kg/day for a further 5 months, at the end of which time the brains were perfusion-fixed for sectioning and microscopy.

This treatment did not affect liver function, with the serum activities of the liver enzymes γ-glutamyltransferase and glutamate dehydrogenase of both the treated and control affected sheep being within the normal ranges for healthy sheep after 3 months of chronic treatment. Drug treatment did not alter feed consumption, and the increase in body weight of the treated animals of 1.7 kg/mo was not significantly different from 2.1 kg/mo for untreated animals.

Easily measurable plasma and CSF concentrations of the drug resulted, with the 8.2- to 8.3-minute retention time HPLC elution peak being separated from other absorbing components. It had the 250- to 450-nm absorption spectrum of minocycline and was identical in both plasma and CSF samples from the experimental sheep and plasma and CSF spiked with unmodified minocycline (Figure 1), indicating that pharmacologic amounts of the untransformed drug passed through the rumen, were absorbed into serum and were partitioned into the CSF. Rises in plasma and CSF concentrations of minocycline lagged behind the dosing program, and plateau concentrations were not reached until week 25 of treatment while the dose was 25 mg/kg/day. The highest concentrations detected, 2,563 nM and 700 nM, respectively, dropped subsequent to the daily dietary dose being lowered from 25 to 16 mg/kg/day. Mean ± SEM minocycline concentrations over the entire treatment period were 1,000 ± 223 nM in plasma and 390 ± 61 nM in CSF.

**Figure 2:**
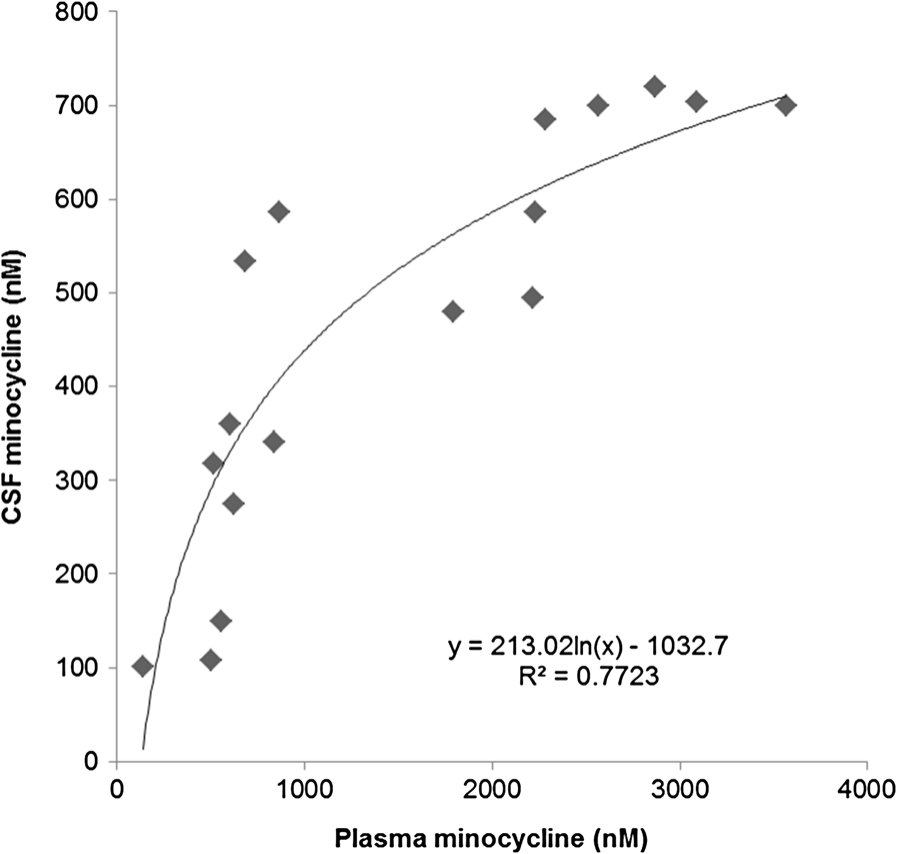
**Minocycline in plasma and cerebrospinal fluid (CSF).** Relationship between minocycline concentrations in plasma and CSF samples taken simultaneously from CLN6 affected sheep chronically treated with minocycline at 25/kg/day.

CSF concentrations of minocycline were always lower than those of matching plasma samples, suggesting that the minocycline entered the brain via the plasma and left via the CSF, but the relationship between them was not linear and a logarithmic function best fitted the data (Figure 2). The CSF-to-plasma concentration ratio was high, up to 0.78, at low plasma concentrations (less than 500 nM) and low, 0.2 to 0.3, at plasma concentrations greater than 1,500 nM. Minocycline distribution was widespread throughout the animals, with the bones, particularly the skull and ribs, being noticeably yellow post-mortem.

### Clinical and pathological effects

Year-long treatment at these pharmacologically relevant concentrations of minocycline in plasma and CSF had no observable effect on the development of symptoms or pathology in the affected sheep. Treatment did not alter the development of blindness. Both the treated and untreated affected sheep scored maximum points for visual awareness up to the age of 9 months, then visual awareness declined to zero in both groups by 15 months of age. The rate of gross brain atrophy was not altered by minocycline treatment either. Brain volumes of both the treated and untreated affected sheep, determined by regular CT scanning, were almost static. At the beginning of the experiments, the brain volume for the treated group was 90.7 ± 6 ml (mean ± SD) compared with 88.5 ± 3 ml for the untreated animals, and these volumes remained virtually unchanged, being 89.6 ± 2 ml and 88.6 ± 2 ml respectively after 15 months. These data are in contrast to the increase in normal sheep brain volumes, from 90 ml to 104 ml, over the same age span.

Neurodegeneration was also assessed by measurements on Nissl-stained sections of the thickness from the pia and the neuron-rich boundary of layers I and II to the easily identifiable boundary between layer V pyramidal neurons and layer VI multiform neurons. These distances were chosen because the layer V–VI is easy to locate, whereas the bottom of layer VI is difficult to identify. Because layer I is the least affected [12], a second measurement, of layers II to V, was also made. These distances were similar in both treated and untreated affected animals and were about 20% less than in normal control brains (Table 1).

**Table 1:** Neurodegeneration and gross neuroinflammation after a year of treatment

	Mean thickness of layers (μm ± SEM)*		
**Layers**	**Treated affected**	**Untreated affected**	**Normal controls**
I–V	1,063 ± 46	995 ± 43	1,285 ± 30
II–V	680 ± 34	728 ± 52	1,043 ± 28
GSB4 positive band	378 ± 29	351 ± 24	–

**Figure 3:**
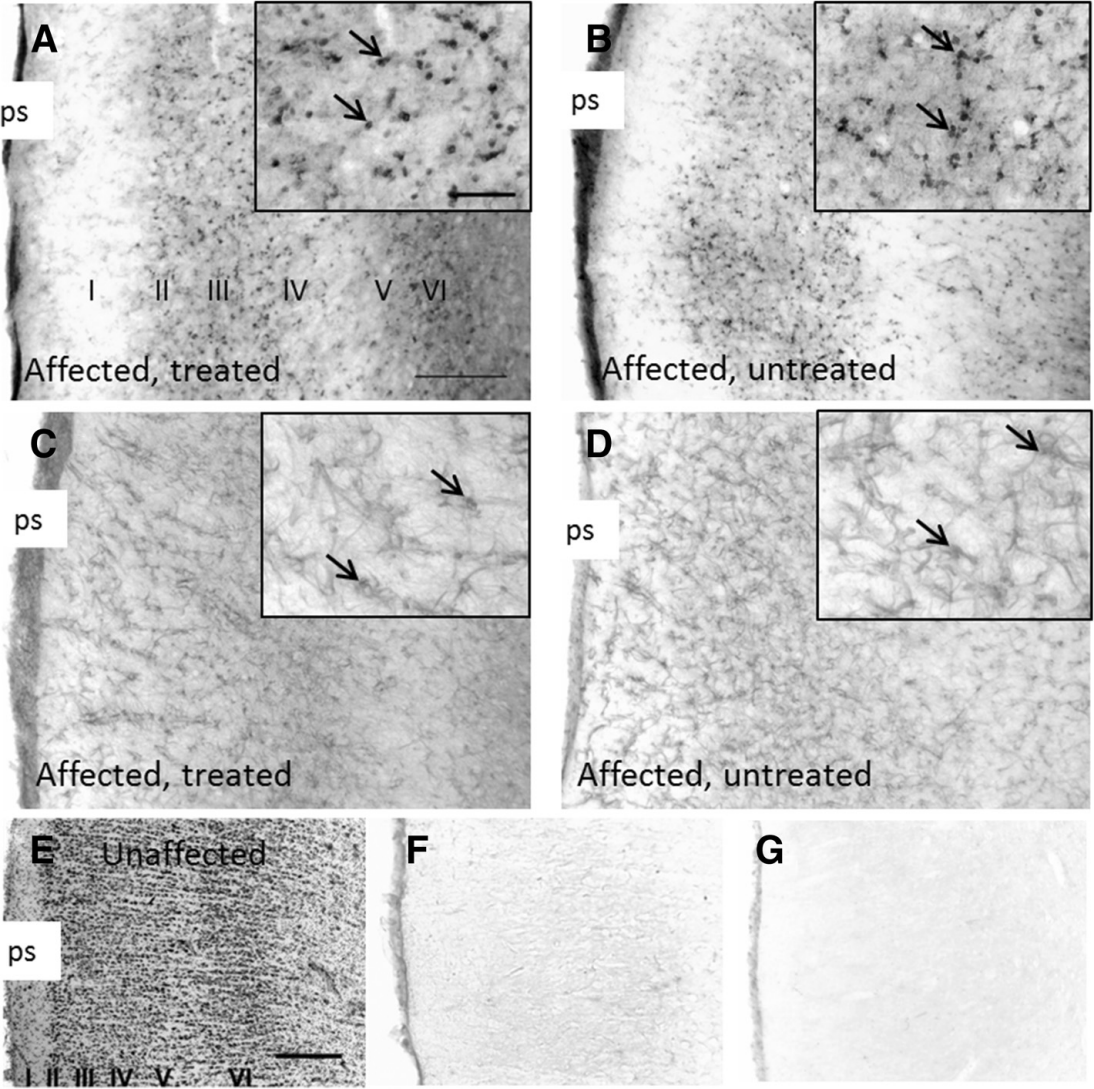
**Glial activation.** Microglial activation and astrocytic activation, as shown by GFAP and GSB4 lectin immunohistochemistry, were not inhibited by chronic minocycline treatment. Sections from the visual cortex of chronically minocycline-treated CLN6 affected sheep stained for microglia with GSB4 lectin **(A)** similarly to sections from affected untreated sheep **(B)**, whereas no staining was observed in the visual cortex of normal brain **(F)**. Sections from the visual cortex of chronically minocycline-treated CLN6 affected sheep stained similarly for astrocytic activation with GFAP **(C)** to sections from affected untreated sheep **(D)**, whereas no staining was observed in the visual cortex of normal brain **(G)**. Nissl staining revealed the cortical layers in the visual cortex of a normal brain **(E)**, indicated by roman numerals and also shown in **(A)**. The pial surface (ps) is to the left in all images. Scale bars on **(A)** through to **(G)** represent 200 μm, and scale bars on the insets represent 50 μm.

*For three animals in each group and a minimum of 25 measurements per brain made in the visual cortex using the layer boundaries as defined in Figures 3A and E. GSB4, biotinylated α-D-galactose-specific isolectin I-B4 from *Griffonia simplicifolia.*

There was also no indication that the chronic minocycline treatment reduced neuroinflammation. Numerous activated amoeboid single microglial cells or clusters of microglia were densely concentrated in bands incorporating cortical layers II and III and layers V and VI of both treated and untreated affected brains (Figure 3). Similar neurodegeneration was also indicated by the similar widths of the GBS4 immunopositive band of cells in the cortex (Table 1). Normal control brains contained no GSB4-reactive microglia. GFAP-positive activated astrocytes bearing thickened and intensely immunostained processes were distributed fairly evenly across the six cortical layers in both the treated and untreated affected brains, but not in unaffected control brains. Quantitative threshold analyses of GSB4 and GFAP confirmed that the treatment had no effect on microglial activation or astrocytosis. Immunoreactivities in the cortical region encompassing neuronal layers II to V in the visual cortex of minocycline-treated and untreated affected animals (± SD, *n* = 3) were 38.0 ± 8.7% and 36.6 ± 6.9%, respectively, for GSB4, and 67.9 ± 12.7% and 63.7 ± 5.4%, respectively, for GFAP.

## Discussion

Sheep became readily accustomed to chronic incremental oral administration of minocycline without any indication of intolerance or ill effects, and, once established, oral administration was simpler and caused fewer concerns than either intraperitoneal or intravenous injection [28]. Any disturbance to the rumen flora, possibly caused by the antibiotic activity of this drug, was transient, and accustomed sheep ate readily and grew at the same rate as controls. The HPLC elution profile and ultraviolet-visible spectra of peaks (Figure 1) gave no indication of minocycline metabolites in either the CSF or sera, including no evidence of the most likely ones, 9-hydroxyminocycline and *N*-demethylated minocycline [29]. Furthermore, sufficient amounts of the drug were circulating to establish concentrations of about 380 nM and 1 μM in the CSF and serum, respectively, a ratio seen in other species [30,31]. On the basis of the brain-to-unbound plasma minocycline ratios of 2.6 to 2.8 reported previously [32,33], the brain concentrations of minocycline in treated sheep were likely to be about 670 nM for the year-long treatment.

Prolonged treatment at this dose did not change the course of the disease, however, despite beginning at an early preclinical age before neurodegeneration had begun. Glial activation commences *in utero*[13] and continues into adulthood in these sheep [12], but loss of cortical neurons does not commence until a little later, at about 5 months of age. The experiments were designed to take advantage of this possible window of opportunity in which suppression of microglial activation might slow or halt the onset of the neurodegeneration and associated disease processes. Treatment started after the animals were weaned at about 3 months of age, but all markers of progress of the disease were unchanged by it. The loss of visual awareness was similar in the treated and affected control animals, as was brain atrophy, with treated and untreated animals losing about 0.4 ml of brain volume per month compared to a gain of 1 ml per month in normal animals. Minocycline did not influence progressive microglial activation or progressive cortical gray matter loss either (Table 1 and Figure 3). This is unlikely to be due to a lack of penetration of minocycline into the brain. It was evident from the yellowing of the bones that the drug was widely distributed in the sheep, and, judging from *in vitro* studies, concentrations of 60 μM to 200 nM should be sufficient [33-35]; thus the CSF concentrations of 380 nM achieved in the current study were expected to be sufficient.

It may be that chronic neuroinflammation does not respond to minocycline inhibition. Normally, reactive gliosis in response to an insult is acute and associated with a cascade of proinflammatory cytokines. Microglia numbers increase and then return to pre-insult numbers [36,37]. A number of studies have shown that induced pro-inflammatory cytokines IL-lα and TNF-α [38-41] decline with minocycline treatment in these situations, but the timing of treatment relative to the initial brain insult may be important. In most instances described, minocycline treatment was commenced prior to experimental insults, [22,38-40,42-44]. Neuroinflammation commences in the second half of gestational life in these sheep [13]; thus, although minocycline treatment began relatively early at weaning, it may have been too late to be effective.

Neuroinflammation in the CLN6 affected sheep is widespread and sustained, and there are mixed reports of anti-inflammatory drugs being effective in these situations [18,45,46], suggesting that the chronic inflammatory process is difficult to subdue or that drugs targeted to the critical steps are required. For instance, high-dose intraperitoneal administration of minocycline resulted in an 80% to 90% decline of pro-inflammatory cytokines after a *Staphylococcus aureus* insult, but no reduction of microglial activation [47].

These data highlight the complexity of the neuroinflammatory cascade and of targeting suppression of it. Apart from the observation of microglial activation and astrocytosis, little is known of the inflammatory pathway in Batten disease, and it is unclear what triggers neuroinflammation in these sheep. It is unlikely that dead and dying neurons are the trigger, as neuronal loss only starts at 5 months of age [12], some 6 months after the first observation of activated cortical glia [13], and the abnormal accumulation of storage bodies containing subunit c is also unlikely to be the trigger. These accumulate ubiquitously throughout the brain, whereas the neuroinflammation is highly regional. Some areas of the brain, such as the hypothalamus, have extensive storage body accumulation but exhibit no neuroinflammation, even at advanced disease stages [48]. It is also now clear from work in other animal models of different forms of Batten disease that neuron loss is invariably preceded by localized glial activation [49], also noted in two canine NCL models [50] and a mouse model of neuronopathic Gaucher disease [51]. By extension, it is unlikely that minocycline treatment alone will be effective for the treatment of these other forms of Batten disease.

It is clear from the above discussion that more needs to be known of the mechanism of the glial activation in the NCLs and which particular parts of the inflammation pathways are critically involved. Studies are underway to address this by determining the regional distribution and activities of cytokines and other inflammation-linked processes in the brains of affected sheep over time. Greater knowledge of the mechanism of action of anti-inflammatory agents would also enhance targeting of therapies by indicating which drugs might be effective. Presently, it is not clear where minocycline acts. A number of studies in mice have shown that induced proinflammatory cytokines such as TNF-α decline with minocycline treatment [38,41], perhaps by inhibition of the p38 mitogen-activated protein kinase (p38 MAPK) pathway. Among other possibilities suggested for minocycline action are down-regulation of major histocompatibility complex II expression through interferon regulatory factor 1 (IRF-1) and protein kinase C α/βII (PKC_α/βII_) inhibition [52], inhibition of secretory phospholipase A_2_ indicated by structural studies [53], neuroprotection by direct scavenging of peroxynitrite [54] and reduction of several other inflammatory factors [55].

## Conclusion

This study has established that oral administration is an effective route for drug delivery to the CNS in ruminants as well as the utility of large animal model studies, which should precede highly speculative procedures in already compromised humans. A case in point is the recent abandonment of minocycline treatment of human Huntington’s disease that had been encouraged by positive results of murine studies [56]. However, this does not preclude a role for anti-inflammatory neuroprotection in the treatment of the NCLs with correctly targeted drugs alone or in combination with other treatments, such as gene therapy. More precise knowledge of the critical steps in the neuroinflammatory cascade and targeting of specific drugs to inhibit them will greatly increase the likelihood of success.

## Abbreviations

CSF: Cerebrospinal fluid; CT: Computed tomography; GSB4: α-D-galactose-specific isolectin I-B4 from *Griffonia simplicifolia*; GFAP: Glial fibrillary acidic protein; HPLC: High-performance liquid chromatography; IL-1α: Interleukin lα; p38 MAPK: p38 mitogen-activated protein kinase; TNF-α: Tumor necrosis factor α.

## Competing interests

The authors declare that they have no competing interests.

## Authors’ contributions

GWK carried out or supervised the experimental work and provided draft material for the MS. DNP organized the funding and resources, managed the project, and wrote and produced the MS. Both authors read and approved the final manuscript.

## References

[B1] Rider JA Rider DL Batten disease: past, present, and future Am J Med Genet 1988 Suppl 5 21 26 10.1002/ajmg.1320310606 3146319

[B2] Kousi M Lehesjoki AE Mole SE Update of the mutation spectrum and clinical correlations of over 360 mutations in eight genes that underlie the neuronal ceroid lipofuscinoses Hum Mutat 2012 33 42 63 10.1002/humu.21624 21990111

[B3] Bras J Verloes A Schneider SA Mole SE Guerreiro RJ Mutation of the parkinsonism gene ATP13A2 causes neuronal ceroid-lipofuscinosis Hum Mol Genet 2012 21 2646 2650 10.1093/hmg/dds089 22388936 PMC3363329

[B4] Nosková L Stránecký V Hartmannová H Přistoupilová A Barešová V Ivánek R Hůlková H Jahnová H van der Zee J Staropoli JF Sims KB Tyynelä J Van Broeckhoven C Nijssen PC Mole SE Elleder M Kmoch S Mutations in *DNAJC5*, encoding cysteine-string protein alpha, cause autosomal-dominant adult-onset neuronal ceroid lipofuscinosis Am J Hum Genet 2011 89 241 252 10.1016/j.ajhg.2011.07.003 21820099 PMC3155175

[B5] Smith KR Dah HHM Canafoglia L Andermann E Damiano J Morbin M Bruni AC Giaccone G Cossette P Saftig P Grötzinger J Schwake M Andermann F Staropoli JF Sims KB Mole SE Franceschetti S Alexander NA Cooper JD Chapman HA Carpenter S Berkovic SF Bahlo M Cathepsin F mutations cause Type B Kufs disease, an adult-onset neuronal ceroid lipofuscinosis Hum Mol Genet 2013 22 1417 1423 10.1093/hmg/dds558 23297359 PMC3596852

[B6] Tammen I Houweling PJ Frugier T Mitchell NL Kay GW Cavanagh JAL Cook RW Raadsma HW Palmer DN A missense mutation (c.184C > T) in ovine *CLN6* causes neuronal ceroid lipofuscinosis in Merino sheep whereas affected South Hampshire sheep have reduced levels of *CLN6* mRNA Biochim Biophys Acta 2006 1762 898 905 10.1016/j.bbadis.2006.09.004 17046213

[B7] Palmer DN Martinus RD Cooper SM Midwinter GG Reid JC Jolly RD Ovine ceroid-lipofuscinosis. The major lipopigment protein and the lipid-binding subunit of mitochondrial ATP synthase have the same NH_2_-terminal sequence J Biol Chem 1989 264 5736 5740 2522438

[B8] Palmer DN Fearnley IM Walker JE Hall NA Lake BD Wolfe LS Haltia M Martinus RD Jolly RD Mitochondrial ATP synthase subunit c storage in the ceroid-lipofuscinoses (Batten disease) Am J Med Genet 1992 42 561 567 10.1002/ajmg.1320420428 1535179

[B9] Chen R Fearnley IM Palmer DN Walker JE Lysine 43 is trimethylated in subunit c from bovine mitochondrial ATP synthase and in storage bodies associated with Batten disease J Biol Chem 2004 279 21883 21887 10.1074/jbc.M402074200 15010464

[B10] Palmer DN Tammen I Drögemüller C Johnson GS Katz ML Lingaas F Mole SE, Williams RE, Goebel HH Large animal models The neuronal ceroid lipofuscinoses (Batten disease) 2011 2 New York: Oxford University Press 284 320

[B11] Oswald MJ Palmer DN Kay GW Barwell KJ Cooper JD Location and connectivity determine GABAergic interneuron survival in the brains of South Hampshire sheep with CLN6 neuronal ceroid lipofuscinosis Neurobiol Dis 2008 32 50 65 10.1016/j.nbd.2008.06.004 18634879 PMC2647510

[B12] Oswald MJ Palmer DN Kay GW Shemilt SJA Rezaie P Cooper JD Glial activation spreads from specific cerebral foci and precedes neurodegeneration in presymptomatic ovine neuronal ceroid lipofuscinosis (CLN6) Neurobiol Dis 2005 20 49 63 10.1016/j.nbd.2005.01.025 16137566

[B13] Kay GW Palmer DN Rezaie P Cooper JD Activation of non-neuronal cells within the prenatal developing brain of sheep with neuronal ceroid lipofuscinosis Brain Pathol 2006 16 110 116 10.1111/j.1750-3639.2006.00002.x 16768750 PMC8096029

[B14] Raivich G Bohatschek M Kloss CUA Werner A Jones LL Kreutzberg GW Neuroglial activation repertoire in the injured brain: graded response, molecular mechanisms and cues to physiological function Brain Res Rev 1999 30 77 105 10.1016/S0165-0173(99)00007-7 10407127

[B15] Stoll G Jander S The role of microglia and macrophages in the pathophysiology of the CNS Prog Neurobiol 1999 58 233 247 10.1016/S0301-0082(98)00083-5 10341362

[B16] Streit WJ Mrak RE Griffin WST Microglia and neuroinflammation: a pathological perspective J Neuroinflammation 2004 1 14 10.1186/1742-2094-1-14 15285801 PMC509427

[B17] Neumann H Control of glial immune function by neurons Glia 2001 36 191 199 10.1002/glia.1108 11596127

[B18] Eikelenboom P Veerhuis R Scheper W Rozemuller AJM van Gool WA Hoozemans JJM The significance of neuroinflammation in understanding Alzheimer’s disease J Neural Transm 2006 113 1685 1695 10.1007/s00702-006-0575-6 17036175

[B19] Kim YS Joh TH Microglia, major player in the brain inflammation: their roles in the pathogenesis of Parkinson’s disease Exp Mol Med 2006 38 333 347 10.1038/emm.2006.40 16953112

[B20] Blum D Chtarto A Tenenbaum L Brotchi J Levivier M Clinical potential of minocycline for neurodegenerative disorders Neurobiol Dis 2004 17 359 366 10.1016/j.nbd.2004.07.012 15571972

[B21] Kim HS Suh YH Minocycline and neurodegenerative diseases Behav Brain Res 2009 196 168 179 10.1016/j.bbr.2008.09.040 18977395

[B22] Yrjänheikki J Keinänen R Pellikka M Hökfelt T Koistinaho J Tetracyclines inhibit microglial activation and are neuroprotective in global brain ischemia Proc Natl Acad Sci (USA) 1998 95 15769 15774 10.1073/pnas.95.26.15769 9861045 PMC28119

[B23] Reinebrant HE Wixey JA Buller KM Disruption of raphé serotonergic neural projections to the cortex: a potential pathway contributing to remote loss of brainstem neurons following neonatal hypoxic-ischemic brain injury Eur J Neurosci 2012 36 3483 3491 10.1111/j.1460-9568.2012.08276.x 22943572

[B24] Kovesdi E Kamnaksh A Wingo D Ahmed F Grunberg NE Long JB Kasper CE Agoston DV Acute minocycline treatment mitigates the symptoms of mild blast-induced traumatic brain injury Front Neurol 2012 3 111 22811676 10.3389/fneur.2012.00111 PMC3397312

[B25] Ferretti MT Allard S Partridge V Ducatenzeiler A Cuello AC Minocycline corrects early, pre-plaque neuroinflammation and inhibits BACE-1 in a transgenic model of Alzheimer’s disease-like amyloid pathology J Neuroinflammation 2012 9 62 10.1186/1742-2094-9-62 22472085 PMC3352127

[B26] Kalonia H Mishra J Kumar A Targeting neuro-inflammatory cytokines and oxidative stress by minocycline attenuates quinolinic-acid-induced Huntington’s disease-like symptoms in rats Neurotox Res 2012 22 310 320 10.1007/s12640-012-9315-x 22392362

[B27] Pijpers A Schoevers EJ Haagsma N Verheijden JH Plasma levels of oxytetracycline, doxycycline, and minocycline in pigs after oral administration in feed J Anim Sci 1991 69 4512 4522 1752826 10.2527/1991.69114512x

[B28] Fagan SC Edwards DJ Borlongan CV Xu L Arora A Feuerstein G Hess DC Optimal delivery of minocycline to the brain: implication for human studies of acute neuroprotection Expt Neurol 2004 186 248 251 10.1016/j.expneurol.2003.12.006 15026261

[B29] Böcker RH Peter R Machbert G Bauer W Identification and determination of the two principal metabolites of minocycline in humans J Chromatogr 1991 568 363 374 1783642 10.1016/0378-4347(91)80174-b

[B30] Agwuh KN MacGowan A Pharmacokinetics and pharmacodynamics of the tetracyclines including glycylcyclines J Antimicrob Chemother 2006 58 256 265 10.1093/jac/dkl224 16816396

[B31] Barza M Brown RB Shanks C Gamble C Weinstein L Relation between lipophilicity and pharmacological behaviour of minocycline, doxycycline, tetracycline, and oxytetracycline in dogs Antimicrob Agents Chemother 1975 8 713 720 10.1128/AAC.8.6.713 1211925 PMC429453

[B32] Aronson AL Pharmacotherapeutics of the newer tetracyclines J Am Vet Med Assoc 1980 176 1061 1068 7216873

[B33] Kremlev SG Roberts RL Palmer C Differential expression of cytokines and chemokine receptors during microglial activation and inhibition J Neuroimmunol 2004 149 1 9 10.1016/j.jneuroim.2003.11.012 15020059

[B34] Tikka TM Koistinaho JE Minocycline provides neuroprotection against *N*-methyl-D-aspartate neurotoxicity by inhibiting microglia J Immunol 2001 166 7527 7533 11390507 10.4049/jimmunol.166.12.7527

[B35] Wang AL Yu ACH Lau LT Lee C Wu LM Zhu XA Tso MOM Minocycline inhibits LPS-induced retinal microglia activation Neurochem Internat 2005 47 152 158 10.1016/j.neuint.2005.04.018 15904993

[B36] Graeber MB Streit WJ Microglia: biology and pathology Acta Neuropathol 2010 119 89 105 10.1007/s00401-009-0622-0 20012873

[B37] Streit WJ Xue QS Life and death of microglia J Neuroimmune Pharmacol 2009 4 371 379 10.1007/s11481-009-9163-5 19680817

[B38] Sriram K Miller DB O’Callaghan JP Minocycline attenuates microglial activation but fails to mitigate striatal dopaminergic neurotoxicity: role of tumor necrosis factor-α J Neurochem 2006 96 706 718 10.1111/j.1471-4159.2005.03566.x 16405514

[B39] Tomás-Camardiel M Rite I Herrera AJ de Pablos RM Cano J Machado A Venero JL Minocycline reduces the lipopolysaccharide-induced inflammatory reaction, peroxynitrite-mediated nitration of proteins, disruption of the blood–brain barrier, and damage in the nigral dopaminergic system Neurobiol Dis 2004 16 190 201 10.1016/j.nbd.2004.01.010 15207276

[B40] Yrjänheikki J Tikka T Keinänen R Goldsteins G Chan PH Koistinaho J A tetracycline derivative, minocycline, reduces inflammation and protects against focal cerebral ischemia with a wide therapeutic window Proc Natl Acad Sci (USA) 1999 96 13496 13500 10.1073/pnas.96.23.13496 10557349 PMC23976

[B41] Zhao C Ling Z Newman MB Bhatia A Carvey PM TNF-α knockout and minocycline treatment attenuates blood–brain barrier leakage in MPTP-treated mice Neurobiol Dis 2007 26 36 46 10.1016/j.nbd.2006.11.012 17234424 PMC1892817

[B42] Henry CJ Huang Y Wynne A Hanke M Himler J Bailey MT Sheridan JF Godbout JP Minocycline attenuates lipopolysaccharide (LPS)-induced neuroinflammation, sickness behaviour and anhedonia J Neuroinflammation 2008 5 15 10.1186/1742-2094-5-15 18477398 PMC2412862

[B43] Wu DC Jackson-Lewis V Vila M Tieu K Teismann P Vadseth C Choi DK Ischiropoulos H Przedborski S Blockade of microglial activation is neuroprotective in the 1-methyl-4-phenyl-1,2,3,6-tetrahydropyridine mouse model of Parkinson disease J Neurosci 2002 22 1763 1771 11880505 10.1523/JNEUROSCI.22-05-01763.2002 PMC6758858

[B44] Boger HA Middaugh LD Granholm AC McGinty JF Minocycline restores striatal tyrosine hydroxylase in GDNF heterozygous mice but not in methamphetamine-treated mice Neurobiol Dis 2009 33 459 466 10.1016/j.nbd.2008.11.013 19110059 PMC2645227

[B45] McGeer PL McGeer EG NSAIDs and Alzheimer disease: epidemiological, animal model and clinical studies Neurobiol Aging 2006 28 639 647 16697488 10.1016/j.neurobiolaging.2006.03.013

[B46] Tuppo EE Arias HR The role of inflammation in Alzheimer’s disease Internat J Biochem Cell Biol 2005 37 289 305 10.1016/j.biocel.2004.07.009 15474976

[B47] Kielian T Esen N Lui S Phulwani NK Syed MM Phillips N Nishina K Cheung AL Schwartzman JD Ruhe JJ Minocycline modulates neuroinflammation independently of its antimicrobial activity in *Staphylococcus aureus*-induced brain abscess Am J Pathol 2007 171 1199 1214 10.2353/ajpath.2007.070231 17717149 PMC1988870

[B48] Kay GW Jay NP Palmer DN The specific loss of GnRH-positive neurons from the hypothalamus of sheep with CLN6 neuronal ceroid lipofuscinosis occurs without glial activation and has only minor effects on reproduction Neurobiol Dis 2011 41 614 623 10.1016/j.nbd.2010.11.008 21111820

[B49] Cooper JD The neuronal ceroid lipofuscinoses: the same, but different? Biochem Soc Trans 2010 38 1448 1452 10.1042/BST0381448 21118105

[B50] Nibe K Nakayama H Uchida K Comparative study of cerebellar degeneration in canine neuroaxonal dystrophy, cerebellar cortical abiotrophy, and neuronal ceroid-lipofuscinosis J Vet Med Sci 2010 72 1495 1499 10.1292/jvms.10-0072 20585192

[B51] Farfel-Becker T Vitner EB Pressey SNR Eilam R Cooper JD Futerman AH Spatial and temporal correlation between neuron loss and neuroinflammation in a mouse model of neuronopathic Gaucher disease Hum Mol Genet 2011 20 1375 1386 10.1093/hmg/ddr019 21252206

[B52] Nikodemova M Watters JJ Jackson SJ Yang SK Duncan ID Minocycline down-regulates MHC II expression in microglia and macrophages through inhibition of IRF-1 and protein kinase C (PKC)_α/βII_ J Biol Chem 2007 282 15208 15216 10.1074/jbc.M611907200 17395590

[B53] Dalm D Palm GJ Aleksandrov A Simonson T Hinrichs W Nonantibiotic properties of tetracyclines: structural basis for inhibition of secretory phopholipase A_2_ J Mol Biol 2010 398 83 96 10.1016/j.jmb.2010.02.049 20211188

[B54] Schildknecht S Pape R Müller N Robotta M Marquardt A Bürkle A Drescher M Leist M Neuroprotection by minocycline caused by direct and specific scavenging of peroxynitrite J Biol Chem 2011 286 4991 5002 10.1074/jbc.M110.169565 21081502 PMC3037611

[B55] Garwood CJ Cooper JD Hanger DP Noble W Anti-inflammatory impact of minocycline in a mouse model of tauopathy Front Psychiatry 2010 1 136 1 8 21423446 10.3389/fpsyt.2010.00136 PMC3059645

[B56] Schwarz H Hickey C Zimmerman C Mazzoni P Moskowitz C Rosas D McCall M Sanchez-Ramos J Perlmutter J Wernle A Higgins D Nickerson C Evans S Kumar R Miracle D Dure L Pendley D Anderson K Cines M Ashizawa T Stanton P Fernandez H Suelter M Leavitt B Decolongon J Cudkowicz M McDermott MP Kieburtz K Marshall F Cha JH Huntington Study Group DOMINO Investigators A futility study of minocycline in Huntington’s disease Mov Disord 2010 25 2219 2224 10.1002/mds.23236 20721920 PMC8801051

